# Safety assessment of a *Mitragyna speciosa* leaf extract by a 90-day feeding study in rats

**DOI:** 10.3389/fphar.2026.1922596

**Published:** 2026-10-21

**Authors:** Paula N. Brown, Michael Chan

**Affiliations:** 1 Natural Health and Food Products Research Group, Centre for Applied Research and Innovation, BC Institute of Technology, Burnaby, BC, Canada; 2 Pharmacognosy Institute and Department of Pharmaceutical Sciences, Retzky College of Pharmacy, University of Illinois Chicago, Chicago, IL, United States

**Keywords:** kratom (Mitragyna speciosa), mitragynine, oral exposure, safety assessment, toxicity

## Abstract

**Background:**

*Mitragyna speciosa* is a deciduous evergreen tree belonging to the coffee (Rubiaceae) family. Traditionally used as a restorative, the extensive historical use data shows kratom has a low risk profile with respect to acute and chronic use. These conclusions cannot be unilaterally extended to all kratom products in the marketplace, of which there is a wide variety, with many far removed from traditional kratom preparations.

**Methods:**

A 90-day toxicity study was conducted for the purpose of identifying a no observed adverse effect level (NOAEL) for a proprietary extract prepared from *M. speciosa* leaf, standardized to a target mitragynine concentration. Sprague Dawley Crl:CD (SD) rats (80 male and 80 female) were grouped as 20 male and 20 female and dosed daily by gavage (10 mL/kg body weight) with SpecioTea™ supplying mitragynine at 0, 15, 30, or 60 mg/kg/day for 90 days. The following parameters and end points were evaluated in this study: clinical signs, body weights, body weight gains, food consumption, ophthalmology, clinical pathology parameters (hematology, coagulation, clinical chemistry, and urinalysis), gross necropsy findings, organ weights, and histopathologic examinations.

**Results:**

Daily oral administration of SpecioTea™ resulted in dose-related reductions in food consumption and lower body weights at ≥15 mg/kg/day. Functional observation battery findings were generally consistent with the known pharmacology of the test article at ≥30 mg/kg/day and non-adverse clinical pathology findings at ≥15 mg/kg/day. Adverse changes were observed in the ovary, testis, and epididymis at 60 mg/kg/day. Thus 30 mg/kg b.w./day of SpecioTea™ was designated as NOAEL.

**Conclusion:**

Due to findings at all SpecioTea™ dose levels, a no-observed effect level (NOEL) could not be established. Based on the observed adverse changes in the ovary, testis, and epididymis at 60 mg/kg/day, the no observed adverse effect level (NOAEL) for SpecioTea™ is 30 mg/kg/day for males and females. The results from this study provides evidence in support of the low toxicity associated with a proprietary kratom leaf extract. Scientifically relevant data from studies such as this should form the basis through which further research on the benefits and safety of kratom products are conducted.

## 1 Introduction

*Mitragyna speciosa* (Korth.) Havil. (Rubiaceae) is a deciduous evergreen tree belonging to the coffee family, that is commonly referred to as Kratom. Growing wild in swampy areas, kratom is native to Thailand, Malaysia, Indonesia, and New Guinea and is also cultivated in these regions (Cinosi et al., 2015; Brown et al., 2017). The leaves of kratom are traditionally consumed as an energizer, relaxant, and to alleviate minor aches and pain (Tanguay, 2011; Brown et al., 2017; Ahmad et al., 2022). Kratom has also been increasingly used to improve mood and quality of life and as substitutes for prescription and illicit opioids for managing pain and opioid withdrawal by people seeking abstinence from opioids (Garcia-Romeu et al., 2020; Chear et al., 2021; Grundmann et al., 2022; Smith et al., 2024; Swogger et al., 2026).

Kratom contains a spectrum of plant secondary metabolites including alkaloids, flavonoids, polyphenolic compounds, terpenes, terpenoids, and secoiridoids (Brown et al., 2017). The alkaloids are recognized as the primary active compounds in kratom*,* with leaves containing several alkaloids representing 0.5%–3.0% of the total weight (Hassan et al., 2013; Kruegel and Grundmann, 2018; Sengnon et al., 2023). The alkaloid, mitragynine, is reported to comprise approximately 51%–82% of the total alkaloids in kratom leaves cultivated in SE Asia (Hassan et al., 2013; Kruegel and Grundmann, 2018; Sharma et al., 2019; Todd et al., 2020; Sengnon et al., 2023). Other major alkaloids found within the leaf include paynantheine, speciogynine and speciociliatine (Sharma et al., 2019; Chear et al., 2021; Manwill et al., 2022; Sengnon et al., 2023). Although initially thought to be present in native kratom leaf, it is now recognized 7-hydroxymitragynine (7-HMG), is a transient human metabolite and an oxidative by-product that has only been observed in post-harvest kratom leaf at trace levels (0.003%–0.04% w/w) (Chear et al., 2021; Zhang et al., 2022; Karunakaran et al., 2024; Brown et al., 2026).

Traditionally kratom leaves are chewed or consumed as a tea or decoction preapred by boiling or steeping dry or fresh leaves for up to an hour before consuming (Wray, 1907; Ahmad and Aziz, 2012; Kamal et al., 2012; Hassan et al., 2013). Reports of traditional intake of kratom leaves by regular daily users in Thailand are in the range of 3–30 leaves per day (Suwanlert, 1975; Assanangkornchai et al., 2007). Given its relative abundance and its purported bioactivities, the amount of mitragynine is used as the major marker when comparing kratom consumption. In a series of recent cross-sectional observational studies in Malayasia, traditionally prepared decoctions were consistently found to contain 21–33 mg mitragynine per serving, with daily consumption of 72–115 mg mitragynine (Singh et al., 2018; Singh et al., 2019).

Available data from extensive history of use shows that kratom, in its traditional form, has a low risk profile with respect to acute and chronic use (Grundmann et al., 2018; Kruegel and Grundmann, 2018; Henningfield et al., 2021; Grundmann et al., 2023). Human clinical studies investigating kratom powder also support these conclusions (Tanna et al., 2022; Huestis et al., 2026; Reissig et al., 2026). Tanna et al. (2022) conducted a pharmacokinetic study involving administration of a single dose of kratom tea prepared by steeping 2 g of kratom powder in 240 mL of 80 °C water (Tanna et al., 2022). The tea was well tolerated and none of the participants experienced any severe adverse events with two particpants reporting light-headedness and mild headache upon placement of the intravenous catheter for blood draw (Tanna et al., 2022). Similar results were reported by Huestis et al. (2026) in a study involving four cohorts of twelve subjects, each receiving one of four increasing doses of 500–4,000 mg of dried kratom leaf powder (Huestis et al., 2026). There were no serious adverse events after administration of test materials or placebo and treatment evident adverse events were generally mild, with a few moderate and three severe adverse events, namely, syncope during blood collection and gastritis (Huestis et al., 2026).

Animal studies investigating mitragynine and kratom extracts have suggested very low toxicity (Kamal et al., 2012; Sabetghadam et al., 2013; Henningfield et al., 2022; Srimangkornkaew et al., 2024; Yuliani et al., 2025). Sabetghadam et al.’s (2013) sub-chronic 28-day study in rats exposed to mitragynine at doses of 1, 10, and 100 mg/kg showed no mortality in any of the treatment groups. A study by Kamal, et al. (2012) provided a single oral dose of kratom aqueous extract at 0, 175, 500, or 2000 mg/kg b.w. to rats (n = 10 per group) and reported no mortalities and no LD_50_ could be established. No other significant alterations in biochemical, hematological and morphological parameters signaling toxicity were observed and a NOAEL >2,000 mg/kg bw was estimated (Kamal et al., 2012). Similar findings were reported by Srimangkornkaew et al.’s (2024) in an acute and sub-chronic oral toxicity study using freeze dried Kratom leaf aqueous extract with male and female rats Wister rats (Srimangkornkaew et al., 2024). No animal mortalities occurred and clinical observations did not show any toxicity or changes in physical examination leading the authors to classify the extract into the GHS category 5 with an LD_50_ cutoff at 5,000 – infinity mg/kg body weight and an NOAEL of 1,000 mg/kg body weight, the highest dose used in the sub-chronic toxicity study. (Srimangkornkaew et al., 2024).

Yuliani et al. (2025) conducted an acute toxicity study on Sprague-Dawlley rats with three study materials; a water extract containing 1.8% mitragynine, an ethanol extract containing 7.5% mitragynine and a concentrated alkaloid extract containing 12.2% mitragynine. Animals were observed for toxic effects during the first 30 min after treatment, then periodically until 4 h post treatment and then daily for 14 days. No toxic symptoms or mortality were observed after oral administration of 5,000 mg/kg b.w. of the water and ethanol extracts, leading the authors to declare these extracts non-toxic with expected LD_50_ above 5,000 mg/kg. The alkaloid extract, dosed at 2000 mg/kg b.w., delivering 244 mg mitragynien per kg b.w., showed moderate toxicity and was classified as GHS category 4 with cut-off LD_50_ of 1,000 mg/kg (Yuliani et al., 2025).

Henningfield et al. (2022) conducted a study comparing respiratory effects of mitragynine to oxycodone through single oral doses of 20, 40, 80, 240 or 400 mg/kg b.w. of mitragynine or 6.75, 60 or 150 mg/kg of oxycodone to adult Sprague Dawley rats via oral gavage (Henningfield et al., 2022). Study results showed that mitragynine did not yield any significant dose-related respiratory depressant or life-threatening effects. Only at the highest mitragynine dose were sedative-like effects observed, albeit less severe as compared to oxycodone (Henningfield et al., 2022).

Still, reports of kratom-associated serious adverse events have emerged in the United States (Prozialeck et al., 2019; Li et al., 2023). These incidents are often associated with non-traditional or commercially modified preparations in which alkaloid concentrations greatly exceed those found in traditional ethnopharmacological contexts (Obeng et al., 2020; Todd et al., 2020; Hanapi et al., 2021; Vadiei et al., 2025). In particular, enrichment or synthetic augmentation of 7-hydroxymitragynine has been identified in some products erroneously labelled as kratom (Lydecker et al., 2016; Sharma and McCurdy, 2021; Hill et al., 2025; Brown et al., 2026). Such alterations in alkaloid composition and potency may underlie the higher incidence of toxicity reported in these modern formulations (Obeng et al., 2020; Hanapi et al., 2021; Zuarth Gonzalez et al., 2025). Even with the exclusion of these products, the kratom marketplace consists of a large variety of products with differing contents and levels of kratom constituents. While the available clinical studies provide evidence supporting the safety of traditional kratom formulations, their findings do not necessarily extend to all products on the market. It is imperative that kratom products undergo clinical testing to determine and ensure their safety profiles.

SpecioTea™ is a proprietary aqueous-based extract prepared from *M. speciosa* leaf that is standardized to a target mitragynine concentration of 0.5 mg/mL. The SpecioTea™ extract is prepared using a liquid extraction procedure commonly used for the preparation of herbal teas, infusions, and decoctions. The resulting extract can be diluted to prepare kratom products that mimic traditionally consumed kratom teas and hot water decoctions traditionally ingested in Southeast Asia. Although SpecioTea™ has been formulated to mimic traditional consumed kratom teas, it is still a modern product that is unique from other products and traditional preparations. It is important that modern kratom derived ingredients are independently evaluated to determine their safety and risk. The overall objective of this study was to determine the potential toxicity of SpecioTea™ through a 90-day sub-chronic oral toxicity rat study.

A preliminary investigation on the same test article had shown that once daily oral gavage was well tolerated in rats at levels of mitragynine at 36 and 70 mg/kg/day, with no clinical signs evident and only a slight effect on weight gain at the 70 mg/kg/day dose (unpublished data). For this 90-day study dose levels of SpecioTea™ extract delivering 60, 30 and 15 mg/kg/day of mitragynine were selected in an attempt to produce graded responses to the test article. The highest dose was expected to produce some toxic effects but not excessive lethality that would prevent meaningful evaluation. The mid-dose level was expected to produce minimally toxic effects and the low-dose level no observable indications of toxicity.

Using normalization to body surface area the 60, 30 and 15 mg/kg/day doses are estimated to give a human equivalent dose (HED) of 9.7, 4.8 and 2.4 mg/kg/day or doses of 582, 288, 144 mg/day for a 60 kg adult.

The following parameters and end points were evaluated in this study: clinical signs, body weights, body weight gains, food consumption, ophthalmology, clinical pathology parameters (hematology, coagulation, clinical chemistry, and urinalysis), gross necropsy findings, organ weights, and histopathologic examinations.

## 2 Materials and methods

### 2.1 Decription of test article

The test material is a concentrated form of a proprietary aqueous-based extract prepared in a procedure analogous to the preparation of kratom traditional teas that have been consumed in Southeast Asia for millennia. SpecioTea™ is prepared from dried, ground, kratom leaves and extracted using water acidified with an organic acid. The concentration of mitragynine in the batch of SpecioTea™ used in the study was verified to be 0.5 mg/mL using a previously reported HPLC methodology (Mudge and Brown, 2017). Additionally, the relative abundance of mitragynine and the major alkaloids were determined and the absence of 7-hydroxymitragynine in the material was confirmed. Results of this analysis is summarized in Table 1.

**TABLE 1:** Relative % of Alkaloids in SpecioTea^™^ test article as determined by HPLC.

Relative % of total alkaloids in SpecioTea™ test article				
Mitragynine	Speciociliatine	Paynantheine	Speciogynine	7-Hydroxymitragynine
59.3	0.8	32.1	7.8	Not detected

In order to get a sufficient dose into the rats via oral gavage, the original SpecioTea™ ingredient, standardized to 0.5 mg/mL, had to be further concentrated. The liquid extract was lyophilized to produce a dried concentrated extract that was re-dissolved in reverse osmosis deionization (RODI) water to obtain test sample liquids at 1.5, 3 and 6 mg/mL of mitragynine. The concentration of mitragynine in the liquid extract and all test samples used in the study were verified using the reported HPLC methodology (Mudge and Brown, 2017). The control article, RODI water, was dispensed into daily aliquots, stored in a refrigerator set to maintain 5 °C until use, and dispensed daily for administration to Group 1 control animals. The aliquots were removed from the refrigerator for at least 30 min before dosing. The control article was stirred continuously during dosing.

### 2.2 Animals and husbandry

For the 90-day study, 88 male and 88 female Sprague DawleyCrl:CD(SD) rats were received from Charles River Laboratories, Raleigh, NC. The animals were 8 weeks old and weighed between 223 and 270 g for males and 163 and 207 g for females at initiation of dosing. Each animal was identified by a cage card and a subcutaneously implanted electronic identification chip. The animals were acclimated to their designated housing for 7 days before the first day of dosing.

Animals were individually housed in polycarbonate cages containing appropriate bedding equipped with an automatic watering valve. Housing and care were as specified in the USDA Animal Welfare Act (9 CFR, Parts 1, 2, and 3) and as described in the *Guide for the Care and Use of Laboratory Animals* from the National Research Council (National Research Council of the National Academies, 2011). Water bottles and water gel were provided, as needed. Each cage was clearly labeled with a color-coded cage card indicating study, group, animals numbers, and sex. Cages were arranged on the racks in group order. Temperatures of 67°F–72°F (19 °C–22 °C) with a relative humidity of 51%–64% were maintained. Temperatures of 68°F–72°F (20 °C–22 °C) with a relative humidity of 30%–54% were maintained in the FOB room. A 12-h light/12-h dark cycle was maintained, except when interrupted for designated procedures. Ten or greater air changes per hour with 100% fresh air (no air recirculation) were maintained in the animal rooms.

PMI Nutrition International Certified Rodent Chow No. 5CR4 was provided *ad libitum* throughout the study, except during designated procedures. The same diet in meal form was provided to individual animals as warranted by clinical signs. The feed was analyzed by the supplier for nutritional components and environmental contaminants. Results of the dietary analyses were provided by the supplier and are on file at the Testing Facility. There were no known contaminants in the feed that would interfere with the objectives of the study. Municipal tap water, after treatment by reverse osmosis and ultraviolet irradiation, was freely available to each animal via an automatic watering system, except during designated procedures. Periodic analysis of the water was performed, and results of these analyses are on file at the Testing Facility. There were no known contaminants in the water that could interfere with the outcome of the study.

For psychological/environmental enrichment, animals were provided with items such as a chewing object (nyla bone), except when interrupted by study procedures/activities. As a reward to promote operant conditioning and desired behavior, animals were offered treats (apple slices and yogurt drops).

### 2.3 Experimental design

The 90-day toxicity study was performed at Charles River Laboratories (Spencerville, OH, United States) in compliance with OECD 407 and 408 testing guidelines and in accordance with the U.S. Department of Health and Human Services, Food and Drug Administration (FDA), United States Code of Federal Regulations Title 21, Part 58: Good Laboratory Practice (GLP) for Nonclinical laboratory Studies and as accepted by Regulatory Authorities throughout the European Union (OECD Principles of Good Laboratory Practice), Japan (MHLW), and other countries that are signatories to the OECD mutual Acceptance of Data Agreement. Animals were assigned to four groups by a stratified randomization scheme designed to achieve similar group mean body weights. Males and females were randomized separately and animals in poor health or at extremes of body weight range were not assigned to groups. Each group consisted of 20 male and 20 female rats. Group treatment is summarized in Table 2.

**TABLE 2:** Experimental design.

Group no.	Test article	Nominal mitragynine dose level (mg/kg/day)	Dose volume (mL/kg)	Nominal^a^ mitragynine dose concentration (mg/mL)	Number of animals	
					Main study	
					Males	Females
1	Control article^a^	0	10	0	20	20
2	SpecioTea™	15	10	1.5	20	20
3	SpecioTea™	30	10	3	20	20
4	SpecioTea™	60	10	6	20	20

^a^ Reverse Osmosis Deionized (RODI) water.

Oral route of exposure was selected because this is the intended route of human exposure. A previous 7-day study conducted on the same test article had shown that oral gavage was well tolerated in rats at levels of mitragynine at 36 and 70 mg/kg/day with no clinical signs evident and only a slight effect on weight gain at 70 mg/kg/day resulting in mean body weights that were 7.2% and 5.4% lower in males and females, respectively when compared to controls (unpublished data). The previous study had also revealed no remarkable changes in clinical pathology parameters and gross pathology necropsy findings were limited to thickening and dark foci of the stomach in two 36 mg/kg/day male rats (unpublished data).

For this study, the test and control articles were administered to the appropriate animals by once daily oral gavage from Days 1–91/92. Animals scheduled for euthanasia on Day 92 were dosed on Days 1–91 and animals scheduled for euthanasia on Day 93 were dosed on Days 1–92. The dose volume for each animal was based on the most recent body weight measurement. The doses were given using a syringe with attached gavage cannula. The first day of dosing was designated as Day 1. The dosing formulations were stirred continuously during dose administration.

### 2.4 In-life procedures, observations and measurements

Throughout the study, animals were observed for general health/mortality and moribundity twice daily, once in the morning and once in the afternoon. Cage side observations were performed once daily, beginning Week −1 and throughout the dosing periods; observations were performed 1–3 h post-dose during the dosing period. Cage side observations were not required on the days of detailed clinical observations during the pretest (prior to Day 1) period. The animals were removed from the cage and a detailed clinical observation was performed at least once weekly, beginning Week −1. Food consumption was quantitatively measured for each animal weekly, starting Week −1, throughout the dosing period, except for on the day of scheduled euthanasia. Each animal was weighed at least once weekly, starting Week −1. A fasted body weight was recorded for each animal on the day of necropsy. Terminal body weights were not collected from animals found dead or euthanized moribund.

### 2.5 Functional observational battery (FOB)

Full functional observational battery (FOB) assessments were performed once prior to in-life initiation (Days −4, −3, and −1) and during Week 13 (Days 86, 87, and 88). These assessments included home cage, removal from home cage, open field evaluation, manipulative tests, and motor activity. Motor activity measurements were performed under red lighting to reduce observational disturbance to the animals. Light readings for each enclosure prior to testing ranged from 0.04 to 0.33 foot candles. A white noise generator was activated prior to open field testing to reduce auditory disturbance to the animals. The white-noise sound level in each enclosure ranged from 70 to 76 dB prior to motor activity measurements.

### 2.6 Ophthalmological examinations

Ophthalmological examinations were performed by a board-certified veterinary ophthalmologist prior to in-life initiation (Day −5) and during the last week of the treatment period (Day 91). The ocular examinations were conducted using a hand-held slit lamp and indirect ophthalmoscope. A short-acting mydriatic solution was used to dilate the eyes and facilitate the indirect ocular examinations.

### 2.7 Clinical sample collection

For non-terminal collection (Days 30 and 60), blood was collected from the jugular vein (under isoflurane anesthesia). For terminal collection (Day 92/93) and unscheduled euthanasia animals, blood was collected from the vena cava (under isoflurane anesthesia at gross necropsy). Urine was collected by urine collection cages containing water bottles with ball-bearing sipper tubes. After collection, samples were transferred to the clinical pathology laboratory for processing. Main study animals were fasted overnight before scheduled clinical pathology sample collections, but had access to water *ad libitum*. Samples were collected according to Table 3.

**TABLE 3:** Samples for clinical pathology evaulation

Group	Time point	Hematology	Coagulation	Clinical chemistry	Urinalysis
1 to 4^a^	Day 30	X	-	X	-
1 and 4^b^	Day 60	X	-	X	-
1 to 4^c^	92/93	X	X	X	X
Unscheduled euthanasia (when possible)	Before euthanasia	X	X	X	-

X = sample was collected; - = not applicable.

^a^ Samples were collected from the first 10/sex/group.

^b^ Samples were collected from the last 10/sex/group.

^c^ Blood samples were collected from the first 5/sex/group on each day (a total of 10/sex/group) for those animals scheduled for euthanasia on Day 92/93. For Group 4, only nine males and seven females were bled for the Day 92/93 collection interval.

### 2.8 Hematology and serum biochemical analysis

Blood samples were analyzed for the following parameters: Red blood cell count, Hemoglobin concentration, Hematocrit, Mean corpuscular volume, Red blood cell distribution width, Mean corpuscular hemoglobin concentration, Mean corpuscular hemoglobin, Reticulocyte count (absolute), Platelet count, White blood cell count, Neutrophil count (absolute), Lymphocyte count (absolute), Monocyte count (absolute), Eosinophil count (absolute), Basophil count (absolute), and Large unstrained cells. One blood smear was prepared from each hematology sample. The slide was labeled, stained, and archived. Slide review was only performed on samples that met flagging criteria to confirm accurate hematology analyzer results.

Blood samples were processed for plasma and serum. Blood plasma was analyzed for the following parameters: Activated partial thromboplastin time, Fibrinogen, Prothrombin time, and Sample quality. Blood serum was analyzed for the following parameters: Alanine aminotransferase, Aspartate aminotransferase, Alkaline phosphatase, Gamma glutamyltransferase, Creatine kinase, Total bilirubin, Urea nitrogen, Creatinine, Calcium, Phosphorus, Total protein, Albumin, Globulin (calculated), Albumin/globulin ratio, Glucose, Cholesterol, Triglycerides, Sodium, Potassium, Chloride, and Sample quality.

### 2.9 Urinalysis

Urine samples were processed and analyzed for the following parameters: Color, Appearance/clarity, Specific gravity, Volume, pH, Protein, Glucose, Bilirubin, Ketones, and Blood.

### 2.10 Terminal procedures

Main study animals surviving until scheduled euthanasia were weighed, samples for evaluation of clinical pathology parameters were collected as specified in Table 4 and the animals were euthanized by isoflurane inhalation via vaporizer set up and induction box, followed by exsanguination. Isoflurane vaporizer was set to 3%–4% to induce anesthesia. Once appropriate anesthetic depth was achieved, vaporizer was set to 5% in order to induce death. Euthanasia was confirmed by exsanguination using a cardiac puncture at a 30° angle to the left of the junction formed by the sternal appendix and the last rib followed by blood draw. When possible, the animals were euthanized rotating across dose groups such that similar numbers of animals from each group, including controls, were necropsied at similar times throughout the day. Animals were fasted (overnight) before their scheduled necropsy. A necropsy was conducted for main study animals that died on study, and specified tissues were saved. Animals were refrigerated before necropsy to minimize autolysis.

**TABLE 4:** Summary of organ weight data - terminal euthanasia (Day 92/93).

Organ parameter	Males			Females		
Group	2	3	4	2	3	4
Dose (mg/kg/day)	15	30	60	15	30	60
No. animals per group	20	20	19	19	20	17
Epididymis (No. weighed)	20	20	19	0	0	0
Absolute value	4.2787	3.1958	0.0887	—	—	—
% of body weight	12.26935	13.33193	16.02705	—	—	—
% of brain weight	5.59606	3.92723	1.04653	—	—	—
Adrenal gland (No. weighed)	20	20	19	19	20	17
Absolute value	7.55187	6.83817	14.84604	−3.01073	12.75623	13.70872
% of body weight	16.60435	18.12522	34.84389	3.91110	22.26257	30.78885
% of brain weight	9.46043	7.84604	16.88481	−3.45930	11.89433	14.51019
Pituitary gland (No. weighed)	20	20	19	19	20	17
Absolute value	−3.53437	−2.43191	−4.39620	5.23510	21.00773	20.06210
% of body weight	4.41001	7.89771	11.82044	12.04372	30.74477	38.76044
% of brain weight	−2.16986	−1.41967	−3.08106	4.52687	19.46555	20.71738
Thyroid gland (No. weighed)	20	20	19	19	20	17
Absolute value	−4.83322	9.29204	4.81531	5.83966	2.14090	15.04049
% of body weight	2.17591	20.80310	21.59542	12.63528	10.18621	31.87905
% of brain weight	−3.34484	10.38142	6.08500	5.04420	1.20990	15.71103
Kidney (No. weighed)	20	20	19	19	20	17
Absolute value	−1.1212	−0.5730	−6.1932	0.9402	9.5816	8.7385
% of body weight	6.81324	9.60315	9.31434	7.62535	18.79113	25.35391
% of brain weight	0.23368	0.38479	−4.98721	0.36962	8.59902	9.55788
Liver (No. weighed)	20	20	19	19	20	17
Absolute value	1.8786	9.8100	16.6505	11.2796	24.7446	31.6546
% of body weight	10.50579	21.81088	29.06148	18.43293	35.54931	52.20835
% of brain weight	3.62963	11.06366	11.98292	10.54524	23.57728	32.55597
Ovary (No. weighed)	0	0	0	19	20	17
Absolute value	—	—	—	−7.6010	−15.7462	−32.7435
% of body weight	—	—	—	0.69912	−7.53430	−22.01692
% of brain weight	—	—	—	−7.88754	−16.19475	−32.21314
Testis (No. weighed)	20	20	19	0	0	0
Absolute value	0.5317	2.0926	−1.5188	—	—	—
% of body weight	8.28681	12.51863	14.40288	—	—	—
% of brain weight	1.75705	2.88599	−0.58225	—	—	—
Thymus (No. weighed)	20	20	19	19	20	17
Absolute value	−11.0005	−23.6168	−28.5819	−12.7389	−22.7464	−18.5539
% of body weight	−5.07935	−16.79602	−18.39317	−8.02864	−16.30674	−5.59286
% of brain weight	−10.29249	−23.48362	−28.28682	−13.33369	−23.20494	−17.57245

^a^ All values expressed as percent difference from control group means.

Based upon statistical analysis of group means, values highlighted in bold are significantly different from control group–P ≤ 0.05; refer to data tables for actual significance levels and tests used.

Organs were weighed at necropsy for all scheduled euthanasia animals. Paired organs were weighed together. Organ weight as a percent of body weight (using the terminal body weight) and organ weight as a percent of brain weight were calculated. Representative samples tissues collected from all animals and preserved in 10% neutral buffered formalin, unless otherwise indicated. Tissues were processed at Histo-Scientific Research Laboratories (HSRL), in conjunction with the Charles River Laboratory. All tissues except those for animal identification and bone marrow smears were trimmed, embedded in paraffin, sectioned, mounted on glass slides, and stained with hematoxylin and eosin. Histopathological evaluation was performed by a board-certified veterinary pathologist.

### 2.11 Statistical analysis

All statistical tests were conducted at the 5% significance level. All pairwise comparisons were conducted using two-sided tests and were reported at the 0.1%, 1%, and 5% levels. Numerical data collected on scheduled occasions for the above described variables were analyzed as indicated according to sex and occasion. Descriptive statistics number, mean, and standard deviation (or %CV or SE when deemed appropriate) were reported whenever possible. Inferential statistics were performed when possible, but excluded semi-quantitative data, and any group with less than three observations.

The following pairwise comparisons were made.

Group 2 vs. Group 1
Group 3 vs. Group 1
Group 4 vs. Group 1

Levene’s test was used to assess the homogeneity of group variances. Datasets with at least three groups were compared using an overall one-way ANOVA F-test if Levene’s test was not significant or the Kruskal-Wallis test if it was. If the overall F-test or Kruskal-Wallis test was found to be significant, then the above pairwise comparisons were conducted using Dunnett’s or Dunn’ test, respectively. Datasets with two groups (the designated control group and one other group) were compared using a t-test if Levene’s test was not significant or Wilcoxon Rank-Sum test if findings were signficant.

Parametric and count FOB data were analyzed by One-Way Analysis of Variance (ANOVA). If significance was detected with ANOVA (p < 0.05), pair-wise group comparisons proceeded using the Dunnett’s test (Dunnett, 1955; Dunnett, 1964). Ranked FOB data was analyzed by Kruskal-Wallis non-parametric ANOVA, followed by Dunn’s test, when p < 0.05. Descriptive (categorical) and quantitative FOB data were analyzed by the Multiple Fisher test (Messick, 1957).

## 3 Results

### 3.1 Clinical and ophthalmological examination

There was no SpecioTea™-related mortality. One 60 mg/kg/day male was found dead on Study Day 7 with a perforation in the cranial portion of the esophagus, just below the larynx. One 60 mg/kg/day female was euthanized moribund on Study Day 91 and had a single esophageal perforation on the left side near the thoracic inlet. Both of these deaths were therefore considered to be the result of gavage errors. Two 60 mg/kg females were found dead on Study Day 30 shortly after blood collection for an interim clinical pathology assessment. These deaths were considered to be primarily attributable to complications arising from the blood collection procedure, although a relationship to test article administration cannot be completely ruled out. Finally, one 15 mg/kg/day female was found dead on Day 49. The cause of death for this rat could not be determined, but in the absence of adverse clinical signs or additional deaths in the 15 mg/kg/day and 30 mg/kg/day groups, this death was considered incidental. All other animals survived to scheduled euthanasia.

There were no clinical signs associated with SpecioTea™ administration in males. Unusual clinical signs in 60 mg/kg/day females such as decreased activity, increased vocalization, hunched posture, swollen firm areas, thin appearance, partly closed eyes, abnormal breathing sounds, and labored breathing were found in one or more of three 60 mg/kg/day females. One of these females had a swollen area on the mouth with accompanying findings of increased vocalization, hunched posture, abnormal breathing sounds, labored breathing, decreased fecal output, and reduced fecal size from Days 64–71 with findings of thin appearance on Days 71–78. These signs resolved by Day 85. Shortly prior to scheduled termination, another of these females had findings of decreased activity, hunched posture, partly closed eyes, firm thoracic swelling, decreased fecal output, and reduced fecal size on Days 90 and/or 91. The third of these females had a finding of hunched posture on Day 38.

There were no SpecioTea™-related ophthalmic findings. Most of the observations on Day 91 consisted of findings of corneal opacity or lens opacity and were observed pre-test and/or in controls, and are common background findings observed in rats of this strain and age.

### 3.2 Functional observational battery (FOB)

SpecioTea™-related changes in functional observational battery parameters during the last week of dosing included an increase in involuntary motor movement (i.e., mild tremors in 4/19) score, a decreased tail pinch score, and a decreased hind limb grip strength in 60 mg/kg/day males and increased gait score or gait abnormality, decreased arousal score, decreased rearing score, decreased body temperature, and decreased motor activity in 60 mg/kg/day males and females. In addition, body temperature, arousal, rearing, and the motor activity score were decreased in 30 mg/kg/day males and rearing and the motor activity score were decreased in 30 mg/kg/day females.

### 3.3 Body weight and food consumption

SpecioTea™ administration reduced mean body weight gain at ≥15 mg/kg/day by 10.9%–28.4% in a dose-related manner in males and females from Days 1–91, resulting in mean body weights that were 6.5%–12.5% lower than those of controls on Day 91.

Mean body weight gains from Days 1–91 and final mean body weights were fairly similar in the 15 and 30 mg/kg/day groups. Mean body weight gains in males were 10.9% and 11.8% lower than controls with Day 91 mean body weights that were 7.3% and 8.4% lower that controls, for 15 mg/kg/day and 30 mg/kg/day males, respectively; mean body weight gains in females were 12.7% and 14.4% lower than controls with Day 91 mean body weights that were 6.5% and 6.6% lower than controls, for 15 mg/kg/day and 30 mg/kg/day females, respectively. At 60 mg/kg/day the effect was more pronounced, as mean body weight gains were 18.8% and 28.4% lower than controls and Day 91 mean body weights were 12.4% and 12.5% lower than controls, for males and females, respectively.

SpecioTea™ administration at ≥15 mg/kg/day resulted in a reduction in food consumption that was generally dose related and corresponded to the observed decreases in mean body weight gain. The effect was most pronounced in 60 mg/kg/day males from Days 43–85, when food consumption ranged from 6.2% to 25.0% lower than that of controls, and for 60 mg/kg/day females from Days 8–85, when food consumption ranged from 6.8% to 23.6% lower than that of controls.

### 3.4 Organ weights

There was an increase in thyroid weight (relative to body weight) in males at ≥30 mg/kg/day and in females at 60 mg/kg/day (relative to body, relative to brain). This correlated histologically to minimal to mild thyroid hypertrophy. There was an increase in pituitary gland weight (absolute, relative to body and relative to brain) in females at ≥30 mg/kg/day. This correlated histologically to minimal pituitary hyperplasia. There was an increase in adrenal gland weight (relative to body weight) at ≥15 mg/kg/day and (absolute, relative to body and relative to brain) at 60 mg/kg in males and ≥30 mg/kg/day in females (absolute, relative to body and relative to brain). This correlated histologically to minimal cortical hypertrophy.

There was an increase in liver weight at 15 mg/kg (absolute) and at ≥30 mg/kg/day (absolute, relative to body and relative to brain) in both males and females. This correlated histologically to minimal hepatocellular hypertrophy. An increase in kidney weight was present at ≥15 mg/kg/day in males (relative to body) and at 15 mg/kg/day (relative to body) and ≥30 mg/kg/day (absolute, relative to body and relative to brain) in females. These findings did not have a histologic correlate. There was a decrease in thymic weight at ≥30 mg/kg/day (absolute, relative to brain) in males and at 30 mg/kg/day (absolute, relative to brain) in females. This did not have a histologic correlate.

There was a decrease in ovarian weight at 30 mg/kg/day (absolute) and at 60 mg/kg/day (absolute, relative to body and relative to brain). This correlated histologically in the 60 mg/kg/day group with a reduction in corpora lutea. Testis and epididymis weights were increased at ≥30 mg/kg/day (relative to body). There were no histologic correlates.

No other test article-related organ weight changes were noted. There were other isolated organ weight values that were statistically different from their respective controls, however there were no patterns, trends, or correlating data to suggest these values were toxicologically relevant. Thus, other organ weight differences observed were considered incidental and/or related to difference of sexual maturity and unrelated to administration of SpecioTea™. Organ weight changes are summarized in Table 4.

### 3.5 Blood hematology, coagulation clinical chemistry

There were no adverse SpecioTea™-related effects on hematology or coagulation parameters on Days 30, 60, or 92/93. There were minimal apparent increases (up to 1.07x control) in red cell mass (erythrocytes, hemoglobin, and hematocrit) on Day 30 in males and females at ≥15 mg/kg/day and on Day 92/93 in males at ≥30 mg/kg/day; some of these differences were statistically significant. These changes were generally not accompanied by changes in other red cell indices and were not consistent among the sampling intervals and were therefore not regarded as meaningful. There were other isolated statistically significant differences in hematology or coagulation parameters that were not regarded as SpecioTea™ related because the magnitude of the change was minimal, the change was not observed consistently from interval to interval, or because no dose response was evident.

There were no adverse SpecioTea™-related changes in clinical chemistry parameters on Days 30, 60, and 92/93. Minimal decreases (down to 0.76x) in blood urea nitrogen were observed in females at ≥30 mg/kg/day on Day 30, in males and females (down to 0.74×) at ≥15 mg/kg/day on Day 60, and in males and females (down to 0.70×) at ≥30 mg/kg/day on Day 92/93. In addition there were minimal decreases in glucose (down to 0.86×) in females at ≥30 mg/kg/day on Day 60. These changes were associated with decreased food consumption in these groups.

There were minimal increases in globulin (up to 1.14×) that generally resulted in increases in total protein (up to 1.11×) in males and females at ≥15 mg/kg/day on Day 30, increases in globulin (1.14×) and total protein (1.15×) in females at 60 mg/kg/day on Day 60 along with increases in albumin (1.14×), and increases in globulin (up to 1.14×), albumin (up to 1.10×), and total protein (up to 1.11×) in females at ≥30 mg/kg/day on Day 92/93. There were other isolated statistically significant differences in hematology or coagulation parameters that were not regarded as SpecioTea™ related because the magnitude of the change was minimal, the change was not observed consistently from interval to interval, or because no dose response was evident.

### 3.6 Urinalysis

There were SpecioTea™-related increases in urine volume with corresponding decreases in specific gravity at ≥30 mg/kg/day in males and at 60 mg/kg/day in females on Day 92/93, consistent with a mild diuretic effect. These changes were not regarded as adverse.

### 3.7 Gross pathology

Test article-related gross pathology findings in terminal euthanasia animals were limited to dark discoloration of the thyroid gland. This finding was observed in males and females at doses ≥15 mg/kg/day and correlated histologically with minimal to mild (follicular cell) hypertrophy. Other histologic findings associated with the thyroid include minimal to moderate cellular debris and minimal to mild pigment deposition.

Other gross findings observed were considered incidental, of the nature commonly observed in this strain and age of rat, and/or were of similar incidence in control and treated animals and, therefore, were considered unrelated to administration of SpecioTea™.

### 3.8 Histopathology

The thyroid gland had minimal to mild follicular cell hypertrophy, at ≥15 mg/kg/day in males and females, there was also minimal to moderate cellular debris in the follicular lumina, at 60 mg/kg/day in males and at ≥15 mg/kg/day in females, as well as minimal to mild brown granular pigment deposition in the follicular cells as well as within the colloid at ≥15 mg/kg/day in males and ≥30 mg/kg/day in females.

The pituitary gland had minimal hyperplasia of the pars distalis in ≥15 mg/kg/day females. There was minimal cortical hypertrophy in the adrenal gland in 60 mg/kg/day males and females. A reduction in corpora lutea of the ovary was identified in three 0 mg/kg/day animals, two 15 mg/kg/day animals, eleven 30 mg/kg/day animals and eight 60 mg/kg/day animals. Although this change occurred in both controls and treated animals it occurred with increased frequency in the 30 and 60 mg/kg/day animals and so was considered potentially SpecioTea™ related. There was minimal epithelial hyperplasia of the vagina in 60 mg/kg/day animals. The vaginal change was considered secondary to the ovarian change and therefore was not included in the read-down. Minimal hepatocellular hypertrophy occurred in the liver in 60 mg/kg/day males and females. There was minimal to moderate degeneration/atrophy of the testes in 60 mg/kg/day animals and minimal to mild cellular debris in the epididymis and minimal spermatid depletion in 60 mg/kg/day animals.

Other microscopic findings observed were considered incidental, of the nature commonly observed in this strain and age of rat, and/or were of similar incidence and severity in control and treated animals and, therefore, were considered unrelated to administration of SpecioTea™. A summary of these histopathology findings is presented in Table 5.

**TABLE 5:** Summary of microscopic findings - terminal euthanasia (Day 92/93).

Microscopic parameter	Males				Females			
Group	1	2	3	4	1	2	3	4
Dose (mg/kg/day)	0	15	30	60	0	15	30	60
No. animals examined	20	20	20	19	20	19	20	17
Thyroid (No. examined)	(20)^a^	(19)	(20)	(19)	(20)	(16)	(17)	(17)
Hypertrophy, follicular cell	0	19	19	19	0	16	15	17
Minimal	0	11	9	4	0	14	5	9
Mild	0	8	11	15	0	2	12	8
Cellular debris	0	0	0	11	0	2	10	6
Minimal	0	0	0	11	0	2	4	6
Mild	0	0	0	0	0	0	5	0
Moderate	0	0	0	0	0	0	1	0
Pigment	0	3	7	19	0	0	11	17
Minimal	0	3	7	11	0	0	11	10
Mild	0	0	0	8	0	0	0	7
Gland pituitary (No. examined)	(20)	(20)	(20)	(19)	(20)	(19)	(20)	(17)
Hyperplasia, pars distalis	0	0	0	0	0	1	8	12
Minimal	0	0	0	0	0	1	8	12
Gland adrenal (No. examined)	(20)	(20)	(20)	(19)	(20)	(19)	(20)	(17)
Hypertrophy, cortical	0	0	0	5	0	0	0	5
Minimal	0	0	0	5	0	0	0	5
Ovary (No. examined)	(−)	(−)	(−)	(−)	(20)	(19)	(20)	(17)
Depletion, corpus luteum	—	—	—	—	3	2	11	8
Minimal	—	—	—	—	0	0	0	0
Mild	—	—	—	—	0	0	0	0
Moderate	—	—	—	—	0	0	2	1
Marked	—	—	—	—	3	2	9	7
Vagina (No. examined)	(−)	(−)	(−)	(−)	(20)	(0)	(0)	(17)
Hyperplasia, epithelial	—	—	—	—	0	—	—	5
Minimal	—	—	—	—	0	—	—	5
Liver (No. examined)	(20)	(20)	(20)	(19)	(20)	(19)	(20)	(17)
Hypertrophy, hepatocellular	0	0	0	8	0	0	0	7
Minimal	0	0	0	8	0	0	0	7
Testis (No. examined)	(20)	(20)	(20)	(19)	(−)	(−)	(−)	(−)
Degeneration/atrophy	0	0	0	3	—	—	—	—
Minimal	0	0	0	2	—	—	—	—
Mild	0	0	0	0	—	—	—	—
Moderate	0	0	0	1	—	—	—	—
Epididymis (No. examined)	(20)	(20)	(20)	(19)	(−)	(−)	(−)	(−)
Cellular debris	0	1	0	3	—	—	—	—
Minimal	0	1	0	2	—	—	—	—
Mild	0	0	0	1	—	—	—	—
Depletion, spermatid	0	0	0	1	—	—	—	—
Minimal	0	0	0	1	—	—	—	—

^a^ Numbers in parentheses represent the number of animals with the finding.

## 4 Discussion

SpecioTea™ is a proprietary aqueous-based extract of *M. speciosa* (kratom) leaves, a plant used in Southeast Asia as restorative to boost energy and alleviate minor aches and pains during the workday (Kumarnsit et al., 2006; Harizal et al., 2010; Brown et al., 2017). In this study, SpecioTea™ administration reduced mean body weight gain and food consumption at ≥15 mg/kg/day in a dose-related manner in males and females from Days 1–91, resulting in mean body weights that were 6.5%–12.5% lower than those of controls on Day 91. Similar SpecioTea™-related effects were observed in a preliminary 7-day study conducted in rats at 36 and 70 mg/kg/day (unpublished data).

Sabetghadam et al. (2013) conducted a 28-day study in Sprague Dawley rats with mitragynine (the principal alkaloid in kratom) at doses of 1, 10, or 100 mg/kg/day by oral gavage; in that study, mean body weights and food intake were significantly lower in 100 mg/kg/day male and female rats compared to controls. In another study conducted in Wistar rats with a kratom extract administered at 40 mg/kg/day for 60 days, similar effects of decreased body weight gain, lower body weights, and reduced food and water intake were observed in male and female animals (Kumarnsit et al., 2006). Further, weight loss and decreased appetite have been reported with chronic use of kratom in humans (Singh et al., 2016). In the current study, body differences in SpecioTea™-treated rats relative to control at termination only exceeded 10% at 60 mg/kg/day. At 15 and 30 mg/kg/day, body weight differences ranged from 6% to 8% lower than controls with only minimal reductions in food consumption. Overall reductions in body weight gain appeared to be related to reductions in food consumption, which in turn could be related to either or both a pharmacological effect of MG, and/or appetite suppression from mild irritation of the GI track following gavage administration of the kratom extract. These changes were not associated with any clinical signs indicative of toxicity and were therefore considered non-adverse.

A number of SpecioTea™-related effects were observed in functional observational battery testing conducted during the last week of dosing that appeared to be consistent with the known pharmacological effects of kratom extracts and mitragynine (Kumarnsit et al., 2006; Harizal et al., 2010; Hassan et al., 2013; Sabetghadam et al., 2013; Brown et al., 2017). Such effects included: a decreased tail pinch score in 60 mg/kg/day males (indicative of antinociceptive effects); decreased hind limb grip strength in 60 mg/kg/day males (consistent with skeletal muscle relaxation) (Chittrakarn et al., 2008); decreased body temperature in males at ≥30 mg/kg/day and in females at 60 mg/kg/day; consistent with the reported antipyretic effects of kratom (Hassan et al., 2013). Decreased arousal score, rearing score, and motor activity was observed at 60 mg/kg/day males and females, decreased motor activity score in 30 mg/kg/day males and females, and decreased rearing in 30 mg/kg/day females, consistent with sedative effects observed at high doses of kratom (Singh et al., 2016).

Four 60 mg/kg/day males had recorded clonic involuntary motor movements (i.e., tremors); however, no tremors or other signs were observed during cage-side observations or detailed clinical observations throughout the study. Of the above functional observational battery findings, the only findings of potential concern would be the tremors observed in a few 60 mg/kg/day males. There were no findings at 15 and 30 mg/kg/day in males and females or in 60 mg/kg/day females that would be considered adverse, as the findings were attributed to the pharmacologic properties of the extract and were clearly reversible, and no related or similar findings were observed at other daily or weekly observation intervals.

Several organ weight differences were observed at terminal euthanasia on Day 92/93. The majority of these differences were small in magnitude, many were artifactual differences in the organ to body weight ratios as a consequence of the slightly lower body weights in SpecioTea™-treated rats, and most differences had no corresponding adverse histopathology findings.

The histopathologic findings in the present study included hepatocellular hypertrophy accompanied by increased absolute and relative liver weights and thyroid follicular cell hypertrophy, adaptive non-adverse changes that are consistent with the administration of microsomal enzyme inducers (Lewis et al., 2002). However, the absence of hepatocellular hypertrophy at 15 and 30 mg/kg/day suggests that the thyroid effect is a primary effect rather than a secondary effect. Minimal to mild thyroid follicular cell hypertrophy was observed at ≥15 mg/kg/day, but resulted in no substantial changes in absolute thyroid weight and was not accompanied by proliferative changes; accordingly, this change was considered to be a non-adverse, adaptive change rather than an adverse effect (Capen, 1997; 1998). In addition, thyroid follicular cell hypertrophy is a reversible change (Tani et al., 2004).

Xenobiotic-induced hepatic and thyroid hypertrophy in Sprague-Dawley rats is considered an adaptive response which is known to disappear after cessation of exposure (Hotz et al., 1997). In a previously published study, increased liver weight and hepatocellular hypertrophy were also observed following administration of mitragynine in rats for 28 days (Sabetghadam et al., 2013). As the liver weight increases and minimal hepatocellular hypertrophy in the current study were not accompanied by marked changes in clinical chemistry parameters indicative of hepatocellular damage or dysfunction and there were no histopathology changes indicative of cytotoxicity or cell death, these liver changes were considered to be non-adverse.

Microscopic changes in the thyroid in the present study included pigment deposition at ≥15 mg/kg/day in males and in females at ≥30 mg/kg/day, and minimal cellular debris in males at 60 mg/kg/day and minimal to moderate cellular debris in females at ≥15 mg/kg/day. The pigment deposition change was of minimal to mild severity at 60 mg/kg/day and only of minimal severity at 15 or 30 mg/kg/day and appeared to be consistent with the uptake of a specific constituent(s) of SpecioTea™, and was therefore regarded as non-adverse. Thyroid follicular cell hypertrophy is commonly observed in subchronic and chronic rat toxicity studies (Hotz et al., 1997; Finch et al., 2006; Funk et al., 2017). Due to the close metabolic relationship between the thyroid gland and liver, this finding is usually seen in combination with hepatocellular hypertrophy as hepatic microsomal enzyme induction contributes to thyroid hormone deiodinization, alterations in glucuronide conjugation, and biliary excretion. Increased thyroid hormone excretion produces a compensatory increase in the secretion of thyroid stimulating hormone (TSH) or thyrotropin in the pituitary gland and subsequent thyroid follicular cell hypertrophy or hyperplasia. The development of thyroid tumors via this mechanism is typical for rats but not relevant for humans as adult rats do only have limited amounts of free circulating thyroxine-binding globulin, which is not the case in humans. Finch et al. (2006) showed that male and female Sprague-Dawley rats fed diets containing phenobarbital and/or pyrethrins promoted the formation of thyroid gland follicular cell proliferation via the induction of CYP isozymes and elevated serum TSH levels and the reduction of serum thyroxine (T_4_) or triiodothyronine (T_3_) levels, and on cessation of treatment these effects were essentially reversible in both sexes. Funk et al. (2017) conclusively demonstrated a direct effect of several types of test compounds on the par distalis of the rat thyroid gland by showing the activation of pituitary gland TSH production following metabolic activation in the liver. No proliferative changes were observed in rats in this study and there were no accompaning findings of active areas of necrosis, apoptosis, or degeneration/regeneration and no inflammatory infiltrates present indicating that the cellular debris findings was an adaptive response and was considered as a non-adverse effect (Lewis et al., 2002).

Other microscopic changes included minimal hyperplasia of the pars distalis of the pituitary in females at ≥15 mg/kg/day accompanied by minimally increased absolute and relative pituitary weights at ≥30 mg/kg/day, minimal cortical hypertrophy of the adrenal gland in 60 mg/kg/day males and females accompanied by increased absolute and relative adrenal weights, and minimal vaginal epithelial hyperplasia at 60 mg/kg/day in females. The observed pituitary weight changes and hyperplasia were of minimal severity and there was no evidence of proliferation of mammary epithelium or other indications of hormonal perturbation; accordingly, these changes were regarded as non-adverse. As the observed finding of cortical hypertrophy of the adrenal gland at 60 mg/kg/day and increases in adrenal gland weight were of minimal severity and were not accompanied by changes indicative of cytotoxicity, these changes were considered to be non-adverse.

The finding of vaginal epithelial hyperplasia in 60 mg/kg/day animals was of minimal severity and considered secondary to the observed ovarian changes at 60 mg/kg/day, and was therefore regarded as non-adverse. Adverse findings in the reproductive tract included an increased incidence of moderate to marked depletion of corpora lutea in the ovary at 60 mg/kg/day with a corresponding substantial decrease (>30%) in absolute and relative (to brain) ovarian weight and minimal to moderate degeneration/atrophy of the testis accompanied by minimal to mild cellular debris and spermatid depletion in the epididymis of males at 60 mg/kg/day.

Due to findings at all SpecioTea™ dose levels, a no-observed effect level (NOEL) could not be established. Based on the observed adverse changes in the ovary, testis, and epididymis at 60 mg/kg/day, the no observed adverse effect level (NOAEL) for SpecioTea™ is 30 mg/kg/day for males and females rats. Using normalization to body surface area a NOAEL of 30 mg/kg/day is estimated to give a human equivalent dose (HED) of 4.8 mg/kg/day, corresponding to a dose of 288 mg/day for a 60 kg adult. Compared to traditional.

## 5 Conclusions

Once daily oral administration of SpecioTea™ to rats at levels of 15, 30, and 60 mg/kg/day resulted in dose-related reductions in body weight gain and food consumption and lower body weights at ≥15 mg/kg/day, functional observation battery findings that were generally consistent with the known pharmacology of the test article at ≥30 mg/kg/day, and non-adverse clinical pathology findings at ≥15 mg/kg/day. Adverse changes were observed in the ovary, testis, and epididymis at 60 mg/kg/day. Thus the 30 mg/kg b.w./day dose in this study was designated as the no observed adverse effect level (NOAEL) for males and females. Due to findings at all SpecioTea™ dose levels, a no-observed effect level (NOEL) could not be established.

Although SpecioTea™ has been formulated to mimic traditional consumed kratom teas it is still a modern product that is unique from other products and traditional preparations. The results from this study are specific to the study material, SpecioTea™, and cannot be unilaterally extended to other kratom products in the marketplace. However, these results do provide additional evidence demonstrating the low toxicity associated with a kratom leaf-based product that are dose aligned with traditional preparations. With the wide variety of formats and varying concentrations of constituents, in the market, many of which far removed from traditional kratom preparations, it is important ingredients are independently evaluated to determine their safety and risk. Scientifically relevant data from studies such as this should form the basis through which further research on the benefits and safety of kratom products are conducted and from which the policies which govern kratom should evolve.

## Data Availability

The raw data supporting the conclusions of this article will be made available by the authors, without undue reservation.

## References

[B1] Ahmad K. Aziz Z. (2012). Mitragyna speciosa use in the northern states of Malaysia: a cross-sectional study. J. Ethnopharmacol. 141 (1), 446–450. 10.1016/j.jep.2012.03.009 22440259

[B2] Ahmad I. Prabowo W. C. Arifuddin M. A. Fadraersada J. Indriyanti N. I. Herman H. (2022). Mitragyna species as pharmacological agents: from abuse to promising pharmaceutical products. Life 12 (2), 1–22. 10.3390/life12020193 PMC8878704 35207481

[B3] Assanangkornchai S. Muekthong A. Sam-Angsri N. Pattanasattayawong U. (2007). The use of mitragynine speciosa (“Krathom”), an addictive plant, in Thailand. Subst. Use Misuse 42 (14), 2145–2157. 10.1080/10826080701205869 18097996

[B4] Brown P. N. Lund J. A. Murch S. J. (2017). A botanical, phytochemical and ethnomedicinal review of the genus mitragyna korth: implications for products sold as kratom. J. Ethnopharmacol. 202, 302–325. 10.1016/j.jep.2017.03.020 28330725

[B5] Brown P. N. Chan M. Zhang X. Brendler T. (2026). Elevated 7-hydroxymitragynine levels found in products misbranded as kratom. J. AOAC Int. 109 (1), 124–130. 10.1093/jaoacint/qsaf094 41065466 PMC12733965

[B6] Capen C. C. (1997). Mechanistic data and risk assessment of selected toxic end points of the thyroid gland. Toxicol. Pathol. 25 (1), 39–48. 10.1177/019262339702500109 9061850

[B7] Capen C. C. (1998). Correlation of mechanistic data and histopathology in the evaluation of selected toxic endpoints of the endocrine system. Toxicol. Lett. 102-103, 405–409. 10.1016/s0378-4274(98)00244-6 10022287

[B8] Chear N. J. Leon F. Sharma A. Kanumuri S. R. R. Zwolinski G. Abboud K. A. (2021). Exploring the chemistry of alkaloids from Malaysian Mitragyna speciosa (kratom) and the role of oxindoles on human opioid receptors. J. Nat. Prod. 84 (4), 1034–1043. 10.1021/acs.jnatprod.0c01055 33635670 PMC8693998

[B9] Chittrakarn S. Sawangjaroen K. Prasettho S. Janchawee B. Keawpradub N. (2008). Inhibitory effects of kratom leaf extract (Mitragyna speciosa Korth.) on the rat gastrointestinal tract. J. Ethnopharmacol. 116 (1), 173–178. 10.1016/j.jep.2007.11.032 18191353

[B10] Cinosi E. Martinotti G. Simonato P. Singh D. Demetrovics Z. Roman-Urrestarazu A. (2015). Following “the roots” of kratom (Mitragyna speciosa): the evolution of an enhancer from a traditional use to increase work and productivity in Southeast Asia to a recreational psychoactive drug in western countries. Biomed. Res. Int. 2015, 968786. 10.1155/2015/968786 26640804 PMC4657101

[B11] Dunnett C. W. (1955). A multiple comparison procedure for comparing several treatments with a control. J. Am. Stat. Assoc. 50 (272), 1096–1121. 10.1080/01621459.1955.10501294

[B12] Dunnett C. W. (1964). New tables for multiple comparisons with a control. Biometrics 20 (3), 482–491. 10.2307/2528490

[B13] Finch J. M. Osimitz T. G. Gabriel K. L. Martin T. Henderson W. J. Capen C. C. (2006). A mode of action for induction of thyroid gland tumors by Pyrethrins in the rat. Toxicol. Appl. Pharmacol. 214 (3), 253–262. 10.1016/j.taap.2006.01.009 16626768

[B14] Funk J. Ebeling M. Singer T. Landes C. (2017). Image analysis for TSH mRNA *in situ* hybridization in pituitary glands from rats with thyroid follicular cell hypertrophy after treatment with three different test compounds. Res. Veterinary Sci. 114, 313–321. 10.1016/j.rvsc.2017.06.007 28646742

[B15] Garcia-Romeu A. Cox D. J. Smith K. E. Dunn K. E. Griffiths R. R. (2020). Kratom (Mitragyna speciosa): user demographics, use patterns, and implications for the opioid epidemic. Drug Alcohol Dependence 208, 107849. 10.1016/j.drugalcdep.2020.107849 32029298 PMC7423016

[B16] Grundmann O. Brown P. N. Henningfield J. Swogger M. Walsh Z. (2018). The therapeutic potential of kratom. Addiction 113 (10), 1951–1953. 10.1111/add.14371 29949213

[B17] Grundmann O. Veltri C. A. Morcos D. Knightes D. Smith K. E. Singh D. (2022). Exploring the self-reported motivations of kratom (Mitragyna speciosa Korth.) use: a cross-sectional investigation. Am. J. Drug Alcohol Abuse 48 (4), 433–444. 10.1080/00952990.2022.2041026 35389321

[B18] Grundmann O. Hendrickson R. G. Greenberg M. I. (2023). Kratom: history, pharmacology, current user trends, adverse health effects and potential benefits. Disease-a-Month 69 (6), 101442. 10.1016/j.disamonth.2022.101442 35732553

[B19] Hanapi N. A. Chear N.J.-Y. Azizi J. Yusof S. R. (2021). Kratom alkaloids: interactions with enzymes, receptors, and cellular barriers. Front. Pharmacol. 12, 751656. 10.3389/fphar.2021.751656 34867362 PMC8637859

[B20] Harizal S. N. Mansor S. M. Hasnan J. Tharakan J. K. J. Abdullah J. (2010). Acute toxicity study of the standardized methanolic extract of Mitragyna speciosa Korth in Rodent. J. Ethnopharmacol. 131 (2), 404–409. 10.1016/j.jep.2010.07.013 20643198

[B21] Hassan Z. Muzaimi M. Navaratnam V. Yusoff N. H. Suhaimi F. W. Vadivelu R. (2013). From Kratom to mitragynine and its derivatives: physiological and behavioural effects related to use, abuse, and addiction. Neurosci. Biobehav. Rev. 37 (2), 138–151. 10.1016/j.neubiorev.2012.11.012 23206666

[B22] Henningfield J. E. Wang D. W. Huestis M. A. (2021). Kratom abuse potential 2021: an updated eight factor analysis. Front. Pharmacol. 12, 775073. 10.3389/fphar.2021.775073 35197848 PMC8860177

[B23] Henningfield J. E. Rodricks J. V. Magnuson A. M. Huestis M. A. (2022). Respiratory effects of oral mitragynine and oxycodone in a rodent model. Psychopharmacol. (Berl.) 239 (12), 3793–3804. 10.1007/s00213-022-06244-z 36308562 PMC9671979

[B24] Hill K. Boyer E. Grundmann O. Smith K. E. (2025). De Facto Opioids: characterization of Novel 7-Hydroxymitragynine and Mitragynine Pseudoindoxyl Product Marketing. Drug Alcohol Dependence 272, 112701. 10.1016/j.drugalcdep.2025.112701 40373645 PMC12138786

[B25] Hotz K. J. Wilson A. G. E. Thake D. C. Roloff M. V. Capen C. C. Kronenberg J. M. (1997). Mechanism of thiazopyr-induced effects on thyroid hormone homeostasis in male sprague–dawley rats. Toxicol. Appl. Pharmacol. 142 (1), 133–142. 10.1006/taap.1996.8032 9007042

[B26] Huestis M. A. Bothmer J. Hudzik T. Henningfield J. E. Swift S. (2026). Safety and tolerability of single and multiple daily oral doses of dried kratom leaf powder in a randomized trial in healthy volunteers. Ther. Drug Monit. 48, 665–675. 10.1097/FTD.0000000000001427 41486478 PMC13557462

[B27] Kamal M. S. A. Ghazali A. R. Yahya N. A. Wasiman M. I. Ismail Z. (2012). Acute toxicity study of standardized Mitrgyna speciosa Korth aqueous extract in Sprague Dawley rats. J. Plant Stud. 2 (2), 120–129. 10.5539/jps.v1n2p120

[B28] Karunakaran T. Vicknasingam B. Chawarski M. C. (2024). Phytochemical analysis of water and ethanol liquid extracts prepared using freshly harvested leaves of Mitragyna speciosa (Korth.). Nat. Prod. Res. 39 (10), 1–8. 10.1080/14786419.2024.2362428 38842220

[B29] Kruegel A. C. Grundmann O. (2018). The medicinal chemistry and neuropharmacology of kratom: a preliminary discussion of a promising medicinal plant and analysis of its potential for abuse. Neuropharmacology 134 (Pt A), 108–120. 10.1016/j.neuropharm.2017.08.026 28830758

[B30] Kumarnsit E. Keawpradub N. Nuankaew W. (2006). Acute and long-term effects of alkaloid extract of Mitragyna speciosa on food and water intake and body weight in rats. Fitoterapia 77 (5), 339–345. 10.1016/j.fitote.2006.04.006 16781828

[B31] Lewis R. W. Billington R. Debryune E. Gamer A. Lang B. Carpanini F. (2002). Recognition of adverse and nonadverse effects in toxicity studies. Toxicol. Pathol. 30 (1), 66–74. 10.1080/01926230252824725 11890477

[B32] Li J. Li J.-X. Jiang H. Li M. Chen L. Wang Y.-Y. (2023). Phytochemistry and biological activities of corynanthe alkaloids. Phytochemistry 213, 113786. 10.1016/j.phytochem.2023.113786 37422009

[B33] Lydecker A. G. Sharma A. McCurdy C. R. Avery B. A. Babu K. M. Boyer E. W. (2016). Suspected adulteration of commercial kratom products with 7-hydroxymitragynine. J. Med. Toxicol. 12 (4), 341–349. 10.1007/s13181-016-0588-y 27752985 PMC5135684

[B34] Manwill P. K. Flores-Bocanegra L. Khin M. Raja H. A. Cech N. B. Oberlies N. H. (2022). Kratom (Mitragyna speciosa) validation: quantitative analysis of indole and oxindole alkaloids reveals chemotypes of plants and products. Planta Med. 88 (9–10), 838–857. 10.1055/a-1795-5876 35468648 PMC9343938

[B35] Messick S. (1957). Nonparametric statistics for the behavioral sciences. Sidney Siegel. McGraw-Hill, New York, 1956. 312 pp. $6.50. Science 126(3267), 266. 10.1126/science.126.3267.266.a

[B36] Mudge E. M. Brown P. N. (2017). Determination of mitragynine in Mitragyna speciosa raw materials and finished products by liquid chromatography with UV detection: single-laboratory validation. J. AOAC Int. 100 (1), 18–24. 10.5740/jaoacint.16-0220 27725074

[B37] Obeng S. Kamble S. H. Reeves M. E. Restrepo L. F. Patel A. Behnke M. (2020). Investigation of the adrenergic and opioid binding affinities, metabolic stability, plasma protein binding properties, and functional effects of selected indole-based kratom alkaloids. J. Med. Chem. 63 (1), 433–439. 10.1021/acs.jmedchem.9b01465 31834797 PMC7676998

[B38] Prozialeck W. C. Avery B. A. Boyer E. W. Grundmann O. Henningfield J. E. Kruegel A. C. (2019). Kratom policy: the challenge of balancing therapeutic potential with public safety. Int. J. Drug Policy 70, 70–77. 10.1016/j.drugpo.2019.05.003 31103778 PMC7881941

[B39] Reissig C. J. Chen L. Nallani S. C. Hawkins E. G. Galati S. Bonson K. (2026). A pilot, dose-finding, pharmacodynamic and pharmacokinetic study of orally administered botanical kratom. J. Clin. Psychopharmacol. 46 (4), 386–398. 10.1097/jcp.0000000000002158 41837407

[B40] Sabetghadam A. Ramanathan S. Sasidharan S. Mansor S. M. (2013). Subchronic exposure to mitragynine, the principal alkaloid of Mitragyna speciosa, in rats. J. Ethnopharmacol. 146 (3), 815–823. 10.1016/j.jep.2013.02.008 23422336

[B41] Sengnon N. Vonghirundecha P. Chaichan W. Juengwatanatrakul T. Onthong J. Kitprasong P. (2023). Seasonal and geographic variation in alkaloid content of kratom (Mitragyna speciosa (Korth.) Havil.) from Thailand. Plants (Basel) 12 (4), 949. 10.3390/plants12040949 36840297 PMC9966779

[B42] Sharma A. McCurdy C. R. (2021). Assessing the therapeutic potential and toxicity of Mitragyna speciosa in opioid use disorder. Expert Opin. Drug Metab. Toxicol. 17 (3), 255–257. 10.1080/17425255.2021.1853706 33213215

[B43] Sharma A. Kamble S. H. León F. Chear N. J. King T. I. Berthold E. C. (2019). Simultaneous quantification of ten key Kratom alkaloids in Mitragyna speciosa leaf extracts and commercial products by ultra-performance liquid chromatography-tandem mass spectrometry. Drug Test. Analysis 11 (8), 1162–1171. 10.1002/dta.2604 30997725 PMC7927418

[B44] Singh D. Narayanan S. Vicknasingam B. (2016). Traditional and non-traditional uses of Mitragynine (Kratom): a survey of the literature. Brain Res. Bull. 126 (Pt 1), 41–46. 10.1016/j.brainresbull.2016.05.004 27178014

[B45] Singh D. Muller C. P. Murugaiyah V. Hamid S. B. S. Vicknasingam B. K. Avery B. (2018). Evaluating the hematological and clinical-chemistry parameters of kratom (Mitragyna speciosa) users in Malaysia. J. Ethnopharmacol. 214, 197–206. 10.1016/j.jep.2017.12.017 29248450

[B46] Singh D. Narayanan S. Grundmann O. Dzulkapli E. B. Vicknasingam B. (2019). Effects of kratom (Mitragyna speciosa Korth.) use in regular users. Subst. Use Misuse 54 (14), 2284–2289. 10.1080/10826084.2019.1645178 31347441

[B47] Smith K. E. Rogers J. M. Sharma A. McCurdy C. Weiss S. T. Dunn K. E. (2024). Responses to a “typical” morning dose of kratom in people who use kratom regularly: a direct-observation study. J. Addict. Med. 18 (2), 144–152. 10.1097/adm.0000000000001259 38174871 PMC10939942

[B48] Srimangkornkaew P. Suwannasaroj K. Sirimontaporn A. (2024). Acute and sub-chronic oral toxicity of Kratom leaves aqueous extract in Wistar rats: acute and Sub-chronic oral toxicity of kratom leaves aqueous extract. Bull. Dep. Med. Sci. 66 (2), 186–207.

[B49] Suwanlert S. (1975). A study of kratom eaters in Thailand. Bull. Narcotics 27 (3), 21–27. 1041694

[B50] Swogger M. T. Torrice T. Grundmann O. (2026). “Chapter 3 - Kratom use: a review of patterns and motivations,” in Kratom. Editors Henningfield J. E. Beyer C. E. Raffa R. B. (Academic Press), 21–34.

[B51] Tanguay P. (2011). Kratom in Thailand. Decriminalisation and community control? Transnational institute series on legislative reform of drug policies. 10.2139/ssrn.1908849

[B52] Tani Y. Maronpot R. R. Foley J. F. Haseman J. K. Walker N. J. Nyska A. (2004). Follicular epithelial cell hypertrophy induced by chronic oral administration of 2,3,7,8-tetrachlorodibenzo-p-dioxin in female Harlan Sprague-Dawley rats. Toxicol. Pathol. 32 (1), 41–49. 10.1080/01926230490260952 14713547

[B53] Tanna R. S. Nguyen J. T. Hadi D. L. Manwill P. K. Flores-Bocanegra L. Layton M. E. (2022). Clinical pharmacokinetic assessment of kratom (Mitragyna speciosa), a botanical product with opioid-like effects, in healthy adult participants. Pharmaceutics 14 (3), 620. 10.3390/pharmaceutics14030620 35335999 PMC8950611

[B54] Todd D. A. Kellogg J. J. Wallace E. D. Khin M. Flores-Bocanegra L. Tanna R. S. (2020). Chemical composition and biological effects of kratom (Mitragyna speciosa): *in vitro* studies with implications for efficacy and drug interactions. Sci. Rep. 10 (1), 19158. 10.1038/s41598-020-76119-w 33154449 PMC7645423

[B55] Vadiei N. Evoy K. E. Grundmann O. (2025). The impact of diverse kratom products on use patterns, dependence, and toxicity. Curr. Psychiatry Rep. 27, 584–592. 10.1007/s11920-025-01631-7 40762659

[B56] Wray L. (1907). Biak: an opium substitute. J. Fed. Malay States Museums 2, 53–56.

[B57] Yuliani T. Dewijanti I. D. Priyoatmojo D. Sukirno Dista R. Angelina M. (2025). Comparison of acute oral toxicity of water, ethanol and alkaloid extract of Kratom (Mitragyna speciosa). AIP Conf. Proc. 3323, 020016(1). 10.1063/5.0279340

[B58] Zhang M. Sharma A. León F. Avery B. Kjelgren R. McCurdy C. R. (2022). Plant growth and phytoactive alkaloid synthesis in kratom [Mitragyna speciosa (Korth.)] in response to varying radiance. PLOS One 17 (4), e0259326. 10.1371/journal.pone.0259326 35472200 PMC9041851

[B59] Zuarth Gonzalez J. D. Alexandria R. K. Mukhopadyay S. McCurdy C. R. McMahon L. R. Wilkerson J. L. (2025). Assessment of abuse liability and respiratory effects of mitragynine and 7-hydroxymitragynine in rats. J. Pharmacol. Toxicol. Methods 133, 107624. 10.1016/j.vascn.2025.107624

